# Redetermined structure of diphenyl­phospho­nimidotriphenyl­phospho­rane: location of the hydrogen atoms and analysis of the inter­molecular inter­actions

**DOI:** 10.1107/S1600536811011500

**Published:** 2011-04-07

**Authors:** Richard Betz, Thomas Gerber, Eric Hosten, Henk Schalekamp

**Affiliations:** a Nelson Mandela Metropolitan University, Summerstrand Campus, Department of Chemistry, University Way, Summerstrand, PO Box 77000, Port Elizabeth, 6031, South Africa

## Abstract

The title compound, C_30_H_25_NOP_2_, is a bulky phosphazene derivative. Its previous crystal structure [Cameron *et al.* (1979 ▶). *Acta Cryst.* B**35**, 1373–1377] is confirmed and its H atoms have been located in the present study. The formal P=N double bond is about 0.05 Å shorter than the P—N single bond and the large P=N—P bond angle reflects the steric strain in the mol­ecule. An intra­molecular C—H⋯O inter­action occurs. In the crystal, short C—H⋯O contacts connect the mol­ecules into chains propagating in [011], which are cross-linked *via* C—H⋯π inter­actions, generating a three-dimensional network. Aromatic π–π stacking also occurs [shortest centroid–centroid separation = 3.6012 (11) Å].

## Related literature

For the previous structure determination, see: Cameron *et al.* (1979 ▶). For graph-set analysis of hydrogen bonds, see: Etter *et al.* (1990 ▶); Bernstein *et al.* (1995 ▶).
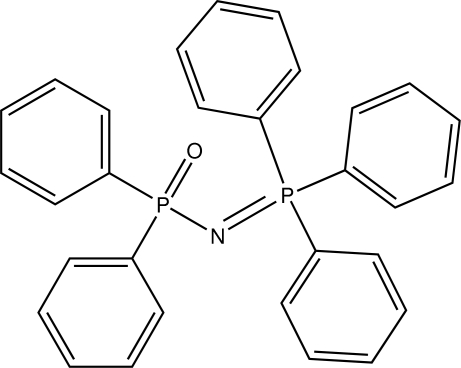


## Experimental

### 

#### Crystal data

C_30_H_25_NOP_2_
*M* *_r_* = 477.45
Orthorhombic, 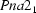

*a* = 17.6607 (12) Å
*b* = 15.1593 (10) Å
*c* = 8.9192 (6) Å
*V* = 2387.9 (3) Å^3^
*Z* = 4
Mo *K*α radiation
μ = 0.21 mm^−1^
*T* = 200 K
0.88 × 0.42 × 0.31 mm

#### Data collection

Bruker APEXII CCD diffractometer
12511 measured reflections
4498 independent reflections
4348 reflections with *I* > 2σ(*I*)
*R*_int_ = 0.021

#### Refinement

*R*[*F* ^2^ > 2σ(*F* ^2^)] = 0.027
*wR*(*F* ^2^) = 0.076
*S* = 1.11
4498 reflections
307 parameters
1 restraint
H-atom parameters constrained
Δρ_max_ = 0.21 e Å^−3^
Δρ_min_ = −0.25 e Å^−3^
Absolute structure: Flack (1983 ▶), 1332 Friedel pairs
Flack parameter: −0.03 (6)

### 

Data collection: *APEX2* (Bruker, 2010 ▶); cell refinement: *SAINT* (Bruker, 2010 ▶); data reduction: *SAINT*; program(s) used to solve structure: *SHELXS97* (Sheldrick, 2008 ▶); program(s) used to refine structure: *SHELXL97* (Sheldrick, 2008 ▶); molecular graphics: *ORTEPIII* (Farrugia, 1997 ▶) and *Mercury* (Macrae *et al.*, 2006 ▶); software used to prepare material for publication: *SHELXL97* and *PLATON* (Spek, 2003 ▶).

## Supplementary Material

Crystal structure: contains datablocks I, global. DOI: 10.1107/S1600536811011500/hb5826sup1.cif

Structure factors: contains datablocks I. DOI: 10.1107/S1600536811011500/hb5826Isup2.hkl

Additional supplementary materials:  crystallographic information; 3D view; checkCIF report

## Figures and Tables

**Table:** 

P1—N1	1.6014 (13)
P2—N1	1.5532 (13)

**Table:** 

P2—N1—P1	146.35 (12)

**Table 2:** Hydrogen-bond geometry (Å, °)

*D*—H⋯*A*	*D*—H	H⋯*A*	*D*⋯*A*	*D*—H⋯*A*
C32—H32⋯O1	0.95	2.34	3.257 (2)	163
C43—H43⋯O1^i^	0.95	2.34	3.257 (2)	162
C45—H45⋯*Cg*1^ii^	0.95	2.92	3.846 (2)	165
C55—H55⋯*Cg*2^iii^	0.95	2.73	3.644 (2)	163

Symmetry codes: (i) 
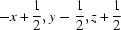
; (ii) 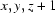; (iii) 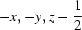.

**Fig. 1.**
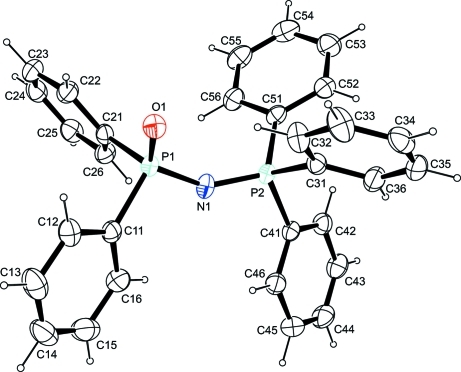
The molecular structure of the title compound, with anisotropic displacement ellipsoids (drawn at 50% probability level).

**Fig. 2.**
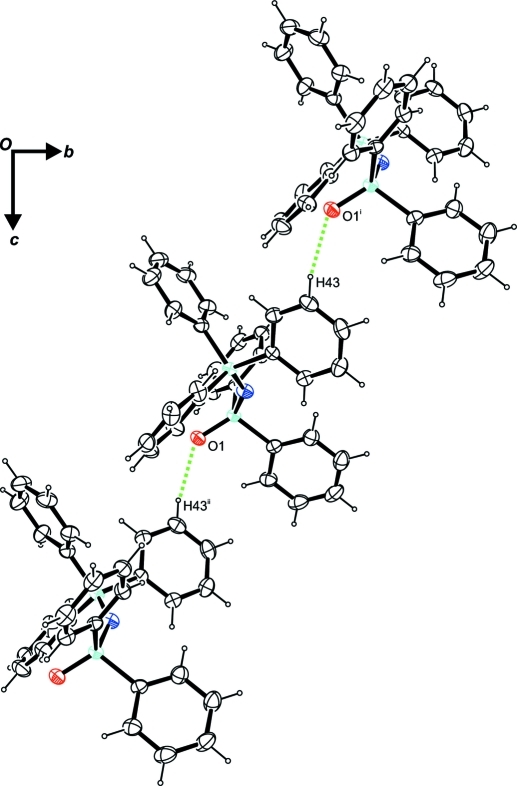
Intermolecular contacts, viewed along [-1 0 0]. Symmetry operators: i -x + 1/2, y - 1/2, z + 1/2; ii -x + 1/2, y + 1/2, z - 1/2.

**Fig. 3.**
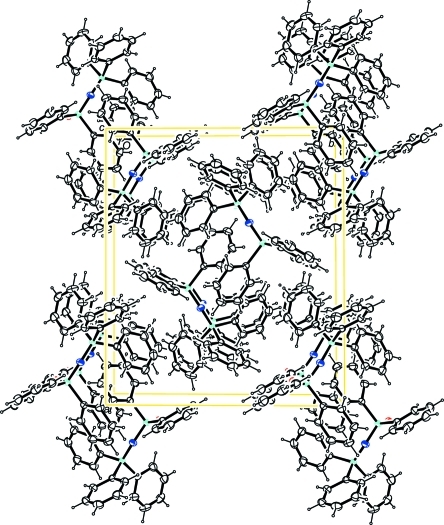
Crystal packing of the title compound, viewed along [0 0 - 1] (anisotropic displacement ellipsoids drawn at 50% probability level).

## supplementary crystallographic information

### Comment

For many main group elements as well as transition and rare earth metals, preferred coordination numbers in coordination compounds are apparent. While coordination numbers of 4, 6 and 8 have been found to be dominant in most cases and, as a consequence, vast structural information has been collected for such compounds in solution and in the solid state, information about other coordination numbers is comparatively limited. Especially for smaller coordination numbers the literature is scant or hitherto completely unknown for many elements. One reason for this certainly is that sometimes challenging synthesis procedures have to be followed and, thus, a general but simple synthetic protocol is desireable. Since such compounds may act as versatile and potent catalysts in many industrial processes and might even show interesting pharmacological properties, we were interested in developing an easy-access-route for their synthesis. Applying bulky ligands might open up a pathway in this aspect. In order to be able to compare metrical parameters in envisioned reaction products, we determined the crystal structure of the title compound. The latter one has already been reported earlier (Cameron *et al.* (1979)), however, no hydrogen atoms were included in the refinement thus ruling out the possibility to assess the role of C–H···*X* contacts.

The length of the N–P bonds deviate by 0.05 Å with the – formal – P–N-double bond found at around 1.55 Å. The P–N–P angle was measured at more than 146 °. The marked widening of this angle in comparison to the value expected for a *sp*^2^-hybridized nitrogen atom can be explained by the repulsive interaction of the phenyl-moieties on both P atoms. The phenyl groups on each phosphorus atom are approximately orientated perpendicular to each other. The least-squares planes defined by their carbon atoms intersect at an angle of 82.19 (6) ° in case of the P(O)Ph_2_-moiety and at angles of 79.82 (5) °, 80.91 (6) ° and 83.28 (6) °, respectively, in case of the PPh_3_-moiety. Due to the formation of an intramolecular C–H···O contact (*see below*), the least-squares plane defined by the P(O)–N–P motif encloses an angle of only 29.40 (9) ° with one of the aromatic carbocycles on the PPh_3_-moiety (Fig. 1). For the same reason, both phenyl groups of the P(O)Ph_2_-moiety adopt a slightly ecliptic conformation with respect to the P(O) motif, the respective dihedral angles were found at about 19 ° and 26 °.

In the crystal structure, intermolecular C–H···O contacts are present whose range falls by more than 0.3 Å below the sum of van-der-Waals radii of the atoms participating. These can be observed between one of the H atoms in *meta*-position of a phenyl group on the PPh_3_-moiety and the O atom of the P(O)Ph_2_-moiety and connect the molecules to infinte chains along [0 1 1] (Fig. 2). Furthermore, intramolecular C–H···O contacts invariably involving hydrogen atoms in *ortho*-position on one of the phenyl groups of the PPh_3_-moiety as well as both phenyl groups of the P(O)Ph_2_-moiety are present. However, the latter two ones are not very pronounced. Additionally, a set of C–H···π contacts are evident involving H atoms and aromatic systems both on the PPh_3_-moiety as well as the P(O)Ph_2_-moiety. Their details are listed in Table 1 (with *C*_g_(1) = C41···C46, *C*_g_(2) = C31···C36 and *C*_g_(3) = C11···C16). In total, the C–H···O contacts as well as the C–H···π contacts connect the molecules to a three dimensional network. In terms of graph-set analysis (Etter *et al.* (1990); Bernstein *et al.* (1995)), the intermolecular C–H···O contacts can be assigned a *C*^1^_1_(8) descriptor on the unitary level while the intramolecular C–H···O contact involving the phenyl group of the PPh_3_-moiety necessitates a *S*^1^_1_(7) descriptor. For the other two intramolecular C–H···O contacts, a *S*^1^_1_(5) each is feasible. An analysis of *C*_g_···*C*_g_ interactions shows the closest distance between two centers of gravity to occur between a phenyl group on the PPh_3_-moiety and a phenyl group on the P(O)Ph_2_-moiety. The distance was measured at 3.6012 (11) Å.

The packing of the title compound in the crystal structure is shown in Figure 3.

### Experimental

The compound was obtained commercially (Aldrich). A colourles block suitable for the X-ray diffraction study were taken directly from the provided material.

### Refinement

Carbon-bound H-atoms were placed in calculated positions (C—H 0.95 Å) and were included in the refinement in the riding model approximation, with *U*(H) set to 1.2*U*_eq_(C).

### Crystal data

**Table:**

C_30_H_25_NOP_2_	*D*_x_ = 1.328 Mg m^−^^3^
*M**_r_* = 477.45	Melting point = 442–445 K
Orthorhombic, *P**n**a*2_1_	Mo *K*α radiation, λ = 0.71073 Å
Hall symbol: P 2c -2n	Cell parameters from 9977 reflections
*a* = 17.6607 (12) Å	θ = 2.7–28.3°
*b* = 15.1593 (10) Å	µ = 0.21 mm^−^^1^
*c* = 8.9192 (6) Å	*T* = 200 K
*V* = 2387.9 (3) Å^3^	Block, colourless
*Z* = 4	0.88 × 0.42 × 0.31 mm
*F*(000) = 1000	

### Data collection

**Table:**

Bruker APEXII CCD diffractometer	4348 reflections with *I* > 2σ(*I*)
Radiation source: fine-focus sealed tube	*R*_int_ = 0.021
graphite	θ_max_ = 28.3°, θ_min_ = 2.9°
φ and ω scans	*h* = −22→23
12511 measured reflections	*k* = −20→16
4498 independent reflections	*l* = −7→11

### Refinement

**Table:**

Refinement on *F*^2^	Secondary atom site location: difference Fourier map
Least-squares matrix: full	Hydrogen site location: inferred from neighbouring sites
*R*[*F*^2^ > 2σ(*F*^2^)] = 0.027	H-atom parameters constrained
*wR*(*F*^2^) = 0.076	*w* = 1/[σ^2^(*F*_o_^2^) + (0.0452*P*)^2^ + 0.5077*P*] where *P* = (*F*_o_^2^ + 2*F*_c_^2^)/3
*S* = 1.11	(Δ/σ)_max_ < 0.001
4498 reflections	Δρ_max_ = 0.21 e Å^−^^3^
307 parameters	Δρ_min_ = −0.25 e Å^−^^3^
1 restraint	Absolute structure: Flack (1983), 1332 Friedel pairs
Primary atom site location: structure-invariant direct methods	Flack parameter: −0.03 (6)

### Fractional atomic coordinates and isotropic or equivalent isotropic displacement parameters (Å^2^)

**Table:**

	*x*	*y*	*z*	*U*_iso_*/*U*_eq_	
P1	0.083912 (19)	0.16050 (2)	0.55082 (5)	0.02138 (9)	
P2	0.227542 (18)	0.05280 (2)	0.53590 (6)	0.02115 (9)	
O1	0.08330 (6)	0.21271 (8)	0.40946 (15)	0.0276 (3)	
N1	0.15673 (7)	0.10297 (9)	0.59418 (19)	0.0272 (3)	
C11	0.06899 (9)	0.23246 (11)	0.7102 (2)	0.0251 (3)	
C12	0.02931 (10)	0.31087 (11)	0.6883 (3)	0.0327 (4)	
H12	0.0132	0.3268	0.5903	0.039*	
C13	0.01304 (11)	0.36602 (13)	0.8084 (3)	0.0420 (5)	
H13	−0.0139	0.4194	0.7924	0.050*	
C14	0.03602 (12)	0.34320 (14)	0.9502 (3)	0.0440 (5)	
H14	0.0252	0.3813	1.0321	0.053*	
C15	0.07484 (12)	0.26496 (15)	0.9752 (3)	0.0426 (5)	
H15	0.0898	0.2489	1.0738	0.051*	
C16	0.09162 (10)	0.21044 (13)	0.8546 (2)	0.0336 (4)	
H16	0.1190	0.1573	0.8710	0.040*	
C21	0.00231 (8)	0.08902 (9)	0.5576 (2)	0.0228 (3)	
C22	−0.05883 (9)	0.10711 (11)	0.4654 (2)	0.0294 (4)	
H22	−0.0563	0.1549	0.3968	0.035*	
C23	−0.12411 (10)	0.05546 (13)	0.4728 (2)	0.0358 (4)	
H23	−0.1663	0.0687	0.4106	0.043*	
C24	−0.12731 (9)	−0.01467 (12)	0.5705 (2)	0.0357 (4)	
H24	−0.1714	−0.0504	0.5741	0.043*	
C25	−0.06683 (10)	−0.03348 (12)	0.6635 (2)	0.0331 (4)	
H25	−0.0693	−0.0819	0.7309	0.040*	
C26	−0.00227 (9)	0.01904 (11)	0.6576 (2)	0.0286 (4)	
H26	0.0391	0.0069	0.7225	0.034*	
C31	0.29089 (9)	0.11812 (11)	0.4214 (2)	0.0250 (3)	
C32	0.26144 (10)	0.18326 (12)	0.3296 (2)	0.0352 (4)	
H32	0.2087	0.1958	0.3315	0.042*	
C33	0.30909 (12)	0.23043 (13)	0.2347 (3)	0.0421 (5)	
H33	0.2887	0.2747	0.1711	0.051*	
C34	0.38560 (11)	0.21312 (13)	0.2327 (2)	0.0378 (4)	
H34	0.4179	0.2455	0.1676	0.045*	
C35	0.41530 (10)	0.14935 (15)	0.3242 (3)	0.0404 (5)	
H35	0.4681	0.1377	0.3222	0.048*	
C36	0.36879 (10)	0.10175 (14)	0.4197 (2)	0.0350 (4)	
H36	0.3898	0.0581	0.4839	0.042*	
C41	0.28149 (8)	0.01741 (10)	0.6966 (2)	0.0222 (3)	
C42	0.31729 (9)	−0.06444 (11)	0.7022 (2)	0.0288 (4)	
H42	0.3146	−0.1031	0.6186	0.035*	
C43	0.35690 (10)	−0.08954 (12)	0.8295 (2)	0.0340 (4)	
H43	0.3809	−0.1456	0.8334	0.041*	
C44	0.36148 (10)	−0.03317 (14)	0.9506 (2)	0.0375 (4)	
H44	0.3886	−0.0504	1.0378	0.045*	
C45	0.32647 (11)	0.04883 (13)	0.9453 (2)	0.0372 (4)	
H45	0.3301	0.0878	1.0284	0.045*	
C46	0.28624 (9)	0.07380 (11)	0.8190 (2)	0.0290 (4)	
H46	0.2618	0.1296	0.8161	0.035*	
C51	0.20882 (9)	−0.04527 (11)	0.4262 (2)	0.0252 (3)	
C52	0.25999 (10)	−0.07830 (13)	0.3230 (2)	0.0335 (4)	
H52	0.3072	−0.0494	0.3088	0.040*	
C53	0.24317 (12)	−0.15319 (13)	0.2399 (3)	0.0402 (5)	
H53	0.2786	−0.1757	0.1695	0.048*	
C54	0.17403 (12)	−0.19490 (12)	0.2607 (3)	0.0396 (5)	
H54	0.1617	−0.2456	0.2030	0.047*	
C55	0.12324 (11)	−0.16321 (12)	0.3645 (3)	0.0369 (4)	
H55	0.0763	−0.1926	0.3792	0.044*	
C56	0.14026 (9)	−0.08887 (11)	0.4474 (2)	0.0310 (4)	
H56	0.1050	−0.0674	0.5192	0.037*	

### Atomic displacement parameters (Å^2^)

**Table:**

	*U*^11^	*U*^22^	*U*^33^	*U*^12^	*U*^13^	*U*^23^
P1	0.01837 (15)	0.02043 (17)	0.0254 (2)	0.00090 (11)	0.00122 (16)	0.00224 (17)
P2	0.01691 (14)	0.02080 (17)	0.0257 (2)	0.00025 (12)	−0.00016 (17)	0.00197 (17)
O1	0.0292 (6)	0.0257 (6)	0.0280 (7)	0.0001 (4)	0.0027 (5)	0.0053 (5)
N1	0.0199 (5)	0.0267 (6)	0.0351 (8)	0.0034 (5)	0.0010 (5)	0.0037 (6)
C11	0.0199 (6)	0.0245 (8)	0.0308 (9)	−0.0035 (6)	0.0018 (6)	−0.0028 (7)
C12	0.0298 (8)	0.0289 (8)	0.0395 (11)	0.0029 (6)	0.0048 (8)	0.0001 (8)
C13	0.0428 (10)	0.0290 (9)	0.0542 (14)	0.0017 (8)	0.0125 (10)	−0.0085 (9)
C14	0.0450 (10)	0.0417 (11)	0.0452 (13)	−0.0110 (8)	0.0144 (10)	−0.0188 (10)
C15	0.0421 (10)	0.0524 (12)	0.0333 (11)	−0.0127 (9)	−0.0004 (9)	−0.0061 (9)
C16	0.0309 (8)	0.0361 (9)	0.0338 (10)	−0.0025 (7)	−0.0022 (8)	−0.0020 (8)
C21	0.0199 (6)	0.0213 (6)	0.0271 (9)	0.0013 (5)	0.0022 (6)	−0.0025 (7)
C22	0.0264 (7)	0.0307 (8)	0.0312 (9)	0.0017 (6)	−0.0034 (7)	−0.0001 (7)
C23	0.0232 (7)	0.0444 (10)	0.0398 (11)	0.0007 (7)	−0.0051 (7)	−0.0080 (9)
C24	0.0265 (7)	0.0418 (9)	0.0388 (11)	−0.0100 (6)	0.0079 (7)	−0.0124 (8)
C25	0.0346 (8)	0.0314 (8)	0.0332 (10)	−0.0077 (7)	0.0073 (8)	0.0006 (8)
C26	0.0258 (7)	0.0317 (8)	0.0282 (9)	−0.0022 (6)	0.0002 (7)	0.0028 (7)
C31	0.0232 (7)	0.0257 (7)	0.0262 (8)	−0.0035 (6)	0.0031 (6)	−0.0017 (7)
C32	0.0318 (8)	0.0326 (9)	0.0411 (12)	0.0050 (7)	0.0094 (8)	0.0071 (8)
C33	0.0481 (10)	0.0323 (9)	0.0460 (12)	0.0030 (8)	0.0174 (10)	0.0110 (9)
C34	0.0417 (10)	0.0398 (10)	0.0320 (10)	−0.0152 (8)	0.0124 (8)	−0.0041 (8)
C35	0.0238 (8)	0.0626 (13)	0.0348 (11)	−0.0106 (8)	0.0025 (8)	−0.0013 (10)
C36	0.0243 (7)	0.0480 (10)	0.0328 (10)	−0.0023 (7)	−0.0012 (7)	0.0056 (8)
C41	0.0186 (6)	0.0234 (7)	0.0247 (8)	−0.0013 (5)	−0.0001 (6)	0.0030 (6)
C42	0.0257 (7)	0.0273 (8)	0.0335 (10)	0.0035 (6)	−0.0004 (7)	−0.0010 (7)
C43	0.0290 (8)	0.0324 (8)	0.0407 (11)	0.0042 (6)	−0.0041 (8)	0.0091 (8)
C44	0.0316 (8)	0.0503 (11)	0.0305 (10)	0.0008 (7)	−0.0061 (7)	0.0094 (9)
C45	0.0346 (9)	0.0451 (10)	0.0319 (10)	−0.0010 (7)	−0.0040 (8)	−0.0076 (9)
C46	0.0255 (7)	0.0279 (8)	0.0335 (10)	0.0004 (6)	−0.0005 (7)	−0.0030 (8)
C51	0.0232 (7)	0.0239 (7)	0.0284 (9)	0.0016 (5)	−0.0052 (6)	0.0014 (6)
C52	0.0289 (8)	0.0379 (9)	0.0339 (11)	0.0013 (7)	−0.0019 (7)	−0.0064 (8)
C53	0.0417 (10)	0.0382 (10)	0.0407 (12)	0.0087 (8)	−0.0027 (9)	−0.0109 (9)
C54	0.0471 (10)	0.0272 (8)	0.0443 (12)	0.0033 (7)	−0.0163 (9)	−0.0032 (8)
C55	0.0346 (8)	0.0282 (8)	0.0480 (12)	−0.0054 (7)	−0.0104 (9)	0.0039 (8)
C56	0.0265 (7)	0.0286 (8)	0.0379 (10)	−0.0019 (6)	−0.0023 (7)	0.0012 (8)

### Geometric parameters (Å, °)

**Table:**

P1—O1	1.4887 (13)	C32—C33	1.392 (3)
P1—N1	1.6014 (13)	C32—H32	0.9500
P1—C21	1.8040 (15)	C33—C34	1.377 (3)
P1—C11	1.8109 (18)	C33—H33	0.9500
P2—N1	1.5532 (13)	C34—C35	1.370 (3)
P2—C41	1.8027 (17)	C34—H34	0.9500
P2—C31	1.8099 (17)	C35—C36	1.386 (3)
P2—C51	1.8099 (17)	C35—H35	0.9500
C11—C16	1.389 (3)	C36—H36	0.9500
C11—C12	1.394 (2)	C41—C46	1.389 (2)
C12—C13	1.389 (3)	C41—C42	1.394 (2)
C12—H12	0.9500	C42—C43	1.387 (3)
C13—C14	1.373 (4)	C42—H42	0.9500
C13—H13	0.9500	C43—C44	1.380 (3)
C14—C15	1.388 (3)	C43—H43	0.9500
C14—H14	0.9500	C44—C45	1.389 (3)
C15—C16	1.388 (3)	C44—H44	0.9500
C15—H15	0.9500	C45—C46	1.384 (3)
C16—H16	0.9500	C45—H45	0.9500
C21—C22	1.385 (2)	C46—H46	0.9500
C21—C26	1.388 (2)	C51—C52	1.384 (3)
C22—C23	1.395 (2)	C51—C56	1.392 (2)
C22—H22	0.9500	C52—C53	1.388 (3)
C23—C24	1.376 (3)	C52—H52	0.9500
C23—H23	0.9500	C53—C54	1.387 (3)
C24—C25	1.382 (3)	C53—H53	0.9500
C24—H24	0.9500	C54—C55	1.375 (3)
C25—C26	1.392 (2)	C54—H54	0.9500
C25—H25	0.9500	C55—C56	1.381 (3)
C26—H26	0.9500	C55—H55	0.9500
C31—C32	1.384 (3)	C56—H56	0.9500
C31—C36	1.398 (2)		
			
O1—P1—N1	120.00 (8)	C31—C32—C33	119.94 (17)
O1—P1—C21	110.00 (8)	C31—C32—H32	120.0
N1—P1—C21	107.83 (7)	C33—C32—H32	120.0
O1—P1—C11	110.08 (7)	C34—C33—C32	120.2 (2)
N1—P1—C11	104.80 (8)	C34—C33—H33	119.9
C21—P1—C11	102.66 (7)	C32—C33—H33	119.9
N1—P2—C41	107.77 (8)	C35—C34—C33	120.16 (18)
N1—P2—C31	114.75 (7)	C35—C34—H34	119.9
C41—P2—C31	106.55 (7)	C33—C34—H34	119.9
N1—P2—C51	115.84 (7)	C34—C35—C36	120.47 (17)
C41—P2—C51	106.37 (8)	C34—C35—H35	119.8
C31—P2—C51	104.91 (8)	C36—C35—H35	119.8
P2—N1—P1	146.35 (12)	C35—C36—C31	119.81 (18)
C16—C11—C12	118.64 (17)	C35—C36—H36	120.1
C16—C11—P1	122.75 (13)	C31—C36—H36	120.1
C12—C11—P1	118.51 (15)	C46—C41—C42	119.50 (16)
C13—C12—C11	120.6 (2)	C46—C41—P2	118.28 (12)
C13—C12—H12	119.7	C42—C41—P2	122.22 (14)
C11—C12—H12	119.7	C43—C42—C41	120.15 (18)
C14—C13—C12	119.86 (19)	C43—C42—H42	119.9
C14—C13—H13	120.1	C41—C42—H42	119.9
C12—C13—H13	120.1	C44—C43—C42	120.04 (17)
C13—C14—C15	120.6 (2)	C44—C43—H43	120.0
C13—C14—H14	119.7	C42—C43—H43	120.0
C15—C14—H14	119.7	C43—C44—C45	120.09 (18)
C14—C15—C16	119.3 (2)	C43—C44—H44	120.0
C14—C15—H15	120.3	C45—C44—H44	120.0
C16—C15—H15	120.3	C46—C45—C44	120.07 (18)
C15—C16—C11	120.92 (18)	C46—C45—H45	120.0
C15—C16—H16	119.5	C44—C45—H45	120.0
C11—C16—H16	119.5	C45—C46—C41	120.15 (16)
C22—C21—C26	119.16 (14)	C45—C46—H46	119.9
C22—C21—P1	118.95 (13)	C41—C46—H46	119.9
C26—C21—P1	121.82 (12)	C52—C51—C56	119.09 (16)
C21—C22—C23	120.35 (17)	C52—C51—P2	122.51 (13)
C21—C22—H22	119.8	C56—C51—P2	118.39 (14)
C23—C22—H22	119.8	C51—C52—C53	120.76 (17)
C24—C23—C22	119.84 (17)	C51—C52—H52	119.6
C24—C23—H23	120.1	C53—C52—H52	119.6
C22—C23—H23	120.1	C54—C53—C52	119.33 (19)
C23—C24—C25	120.51 (15)	C54—C53—H53	120.3
C23—C24—H24	119.7	C52—C53—H53	120.3
C25—C24—H24	119.7	C55—C54—C53	120.31 (18)
C24—C25—C26	119.50 (18)	C55—C54—H54	119.8
C24—C25—H25	120.3	C53—C54—H54	119.8
C26—C25—H25	120.3	C54—C55—C56	120.23 (18)
C21—C26—C25	120.63 (16)	C54—C55—H55	119.9
C21—C26—H26	119.7	C56—C55—H55	119.9
C25—C26—H26	119.7	C55—C56—C51	120.27 (18)
C32—C31—C36	119.36 (16)	C55—C56—H56	119.9
C32—C31—P2	119.46 (12)	C51—C56—H56	119.9
C36—C31—P2	121.14 (14)		
			
C41—P2—N1—P1	173.79 (15)	C41—P2—C31—C36	29.82 (17)
C31—P2—N1—P1	55.30 (19)	C51—P2—C31—C36	−82.72 (17)
C51—P2—N1—P1	−67.26 (19)	C36—C31—C32—C33	1.3 (3)
O1—P1—N1—P2	−25.4 (2)	P2—C31—C32—C33	−176.30 (16)
C21—P1—N1—P2	101.52 (17)	C31—C32—C33—C34	−0.6 (3)
C11—P1—N1—P2	−149.62 (16)	C32—C33—C34—C35	0.0 (3)
O1—P1—C11—C16	−157.41 (13)	C33—C34—C35—C36	−0.1 (3)
N1—P1—C11—C16	−27.08 (16)	C34—C35—C36—C31	0.8 (3)
C21—P1—C11—C16	85.50 (15)	C32—C31—C36—C35	−1.4 (3)
O1—P1—C11—C12	26.37 (15)	P2—C31—C36—C35	176.14 (16)
N1—P1—C11—C12	156.70 (13)	N1—P2—C41—C46	−38.07 (14)
C21—P1—C11—C12	−90.72 (14)	C31—P2—C41—C46	85.57 (13)
C16—C11—C12—C13	0.3 (2)	C51—P2—C41—C46	−162.90 (13)
P1—C11—C12—C13	176.66 (14)	N1—P2—C41—C42	141.06 (13)
C11—C12—C13—C14	−0.2 (3)	C31—P2—C41—C42	−95.30 (14)
C12—C13—C14—C15	−0.6 (3)	C51—P2—C41—C42	16.23 (16)
C13—C14—C15—C16	1.2 (3)	C46—C41—C42—C43	0.5 (2)
C14—C15—C16—C11	−1.1 (3)	P2—C41—C42—C43	−178.67 (13)
C12—C11—C16—C15	0.3 (3)	C41—C42—C43—C44	−0.5 (3)
P1—C11—C16—C15	−175.88 (14)	C42—C43—C44—C45	0.0 (3)
O1—P1—C21—C22	−18.58 (16)	C43—C44—C45—C46	0.7 (3)
N1—P1—C21—C22	−151.12 (14)	C44—C45—C46—C41	−0.8 (3)
C11—P1—C21—C22	98.56 (15)	C42—C41—C46—C45	0.2 (2)
O1—P1—C21—C26	164.52 (14)	P2—C41—C46—C45	179.36 (14)
N1—P1—C21—C26	31.99 (17)	N1—P2—C51—C52	155.63 (15)
C11—P1—C21—C26	−78.33 (15)	C41—P2—C51—C52	−84.66 (17)
C26—C21—C22—C23	−0.1 (3)	C31—P2—C51—C52	28.00 (17)
P1—C21—C22—C23	−177.12 (14)	N1—P2—C51—C56	−24.50 (18)
C21—C22—C23—C24	−1.0 (3)	C41—P2—C51—C56	95.21 (15)
C22—C23—C24—C25	1.2 (3)	C31—P2—C51—C56	−152.12 (14)
C23—C24—C25—C26	−0.1 (3)	C56—C51—C52—C53	0.8 (3)
C22—C21—C26—C25	1.2 (3)	P2—C51—C52—C53	−179.31 (16)
P1—C21—C26—C25	178.09 (14)	C51—C52—C53—C54	0.3 (3)
C24—C25—C26—C21	−1.1 (3)	C52—C53—C54—C55	−1.1 (3)
N1—P2—C31—C32	−33.42 (18)	C53—C54—C55—C56	0.9 (3)
C41—P2—C31—C32	−152.60 (15)	C54—C55—C56—C51	0.2 (3)
C51—P2—C31—C32	94.86 (16)	C52—C51—C56—C55	−1.0 (3)
N1—P2—C31—C36	148.99 (15)	P2—C51—C56—C55	179.07 (15)

### Hydrogen-bond geometry (Å, °)

**Table:**

*D*—H···*A*	*D*—H	H···*A*	*D*···*A*	*D*—H···*A*
C32—H32···O1	0.95	2.34	3.257 (2)	163
C43—H43···O1^i^	0.95	2.34	3.257 (2)	162
C45—H45···Cg1^ii^	0.95	2.92	3.846 (2)	165
C55—H55···Cg2^iii^	0.95	2.73	3.644 (2)	163

Symmetry codes: (i) −*x*+1/2, *y*−1/2, *z*+1/2; (ii) *x*, *y*, *z*+1; (iii) −*x*, −*y*, *z*−1/2.
